# Aversive Learning in Honeybees Revealed by the Olfactory Conditioning of the Sting Extension Reflex

**DOI:** 10.1371/journal.pone.0000288

**Published:** 2007-03-14

**Authors:** Vanina Vergoz, Edith Roussel, Jean-Christophe Sandoz, Martin Giurfa

**Affiliations:** Research Centre on Animal Cognition, Centre National de la Recherche Scientifique (CNRS)-University Paul-Sabatier, Toulouse, France; Freie Universitaet Berlin, Germany

## Abstract

Invertebrates have contributed greatly to our understanding of associative learning because they allow learning protocols to be combined with experimental access to the nervous system. The honeybee *Apis mellifera* constitutes a standard model for the study of appetitive learning and memory since it was shown, almost a century ago, that bees learn to associate different sensory cues with a reward of sugar solution. However, up to now, no study has explored aversive learning in bees in such a way that simultaneous access to its neural bases is granted. Using odorants paired with electric shocks, we conditioned the sting extension reflex, which is exhibited by harnessed bees when subjected to a noxious stimulation. We show that this response can be conditioned so that bees learn to extend their sting in response to the odorant previously punished. Bees also learn to extend the proboscis to one odorant paired with sugar solution and the sting to a different odorant paired with electric shock, thus showing that they can master both appetitive and aversive associations simultaneously. Responding to the appropriate odorant with the appropriate response is possible because two different biogenic amines, octopamine and dopamine subserve appetitive and aversive reinforcement, respectively. While octopamine has been previously shown to substitute for appetitive reinforcement, we demonstrate that blocking of dopaminergic, but not octopaminergic, receptors suppresses aversive learning. Therefore, aversive learning in honeybees can now be accessed both at the behavioral and neural levels, thus opening new research avenues for understanding basic mechanisms of learning and memory.

## Introduction

Associative learning allows extracting the logical structure of the world as it enables making predictions about stimuli and their potential outcomes. Honeybees (*Apis mellifera*) constitute a traditional invertebrate model for the study of associative learning at the behavioral, cellular and molecular levels [1]–[4]. For almost a century, research on honeybee learning and memory has made significant contributions to our general understanding of these processes, but it has focused so far on a single form of learning: appetitive learning, in which bees are rewarded with sucrose on particular stimuli or for performing a given behavior [5]. Since Karl von Frisch, who first discovered the immense potential of this appetitive behavior [6], researchers interested in bee learning have concentrated on appetitive learning. Indeed, a single Pavlovian protocol, the olfactory conditioning of the proboscis extension reflex (PER) [7], [8], has been used for 45 years as the unique tool to access the neural and molecular bases of learning in honeybees. This protocol relies on PER, the appetitive reflex exhibited by a harnessed honeybee to a sugar reward (the unconditioned stimulus or US) delivered to its antennae and mouth parts. After pairing odorant (the conditioned stimulus or CS) and sucrose presentations, the bee learns to associate odorant and sugar reward and therefore extends its proboscis in response to the odorant alone [7], [8].

In contrast, in the fruit fly *Drosophila melanogaster*, the other insect model ubiquitously used in this research field [9]–[11], aversive learning has been the dominant framework. Olfactory learning in fruit flies is generally studied by training flies to avoid an odorant associated with an electric shock in a T-maze [12]. Due to obvious differences in behavioral and motivational contexts, and to the impossibility to equate US nature and strength, caution is needed when comparing appetitive and aversive learning in bees and flies, respectively.

To facilitate such a comparison, we studied aversive learning in honeybees and established a new conditioning protocol for honeybees using the sting extension reflex (SER), which is a defensive response to potentially noxious stimuli [13]. As no appetitive responses are involved in this behavioral context, true aversive learning could be studied in harnessed honeybees. Using odorants paired with electric shocks, we conditioned the SER so that bees learned to extend their sting in response to odorants previously punished. They could also learn to master simultaneously appetitive and aversive associations and exhibited the appropriate response, PER or SER, to the appropriate odorant. We show that aversive learning in honeybees is mediated by dopamine and not by octopamine, which mediates appetitive learning. Our work allows, therefore, accessing for the first time aversive learning in honeybees, both at the behavioral and neural levels.

## Results

**Figure 1:**
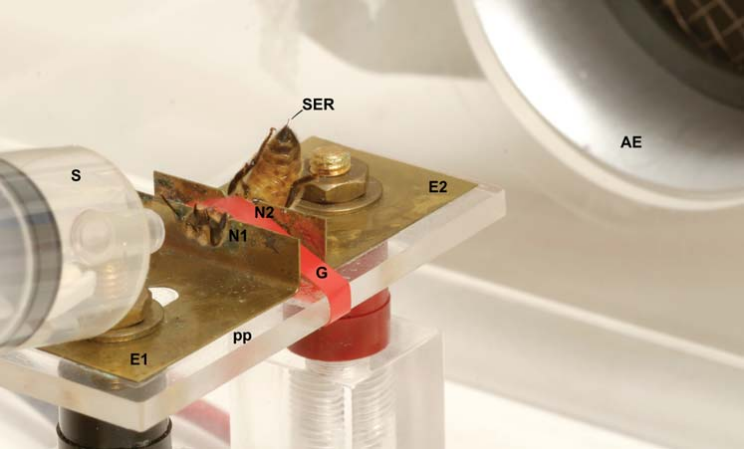
View of a honeybee in the experimental set-up. The bee is fixed between two brass plates (E1, E2) set on a plexiglas plate (pp), with EEG cream smeared on the two notches (N1, N2) to ensure good contact between the plates and the bee, and a girdle (G) that clamped the thorax to restrain mobility. The bee closes a circuit and receives a mild electric shock (7.5V) which induces the sting extension reflex (SER). An originally neutral odorant is delivered through a 20 ml syringe (S) placed 1cm from the antennae. Odorant stimulation lasted 5 sec. The electric shock started 3 sec after odorant onset and lasted 2 sec so that it ended with odorant offset. Contamination with remains of odorants used for conditioning or pheromones is avoided via an air extractor (AE) which is on continuously.

Honeybees were fixed individually on a metallic holder so that they built a bridge between two brass plates through which a 2 sec mild electric shock (7.5 V) was delivered by a stimulator (60 Hz-AC current) (Fig. 1). Bees treated in this way extend their sting in response to the electric shock. This reflex has been previously studied [14]–[17] but so far no study attempted to condition it. The sting extension reflex was rated with a 1 when the sting was visible between the sternal and tergal plaques of the seventh abdominal segment during the 2-sec shock. Absence of response was scored as 0. The intensity of the electric stimulation chosen ensures a reliable unconditioned reaction without physical injury despite repetitive stimulation (see below).

**Figure 2:**
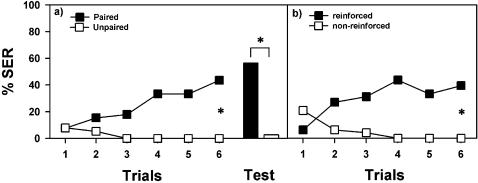
Associative olfactory conditioning of the sting extension reflex (SER) in honeybees. a) Responses (SER) of bees trained with an odorant explicitly paired with an electric shock (black squares; n = 38) and with odorant and unpaired electric shock (white squares; n = 39) during 6 trials. Only the bees in the paired group learned the association and extended their sting as a response to the odorant. One hour after conditioning an olfactory aversive memory was present in the paired (black bar), but not in the unpaired, group (white bar). b) Responses (SER) of bees (n = 48) trained to discriminate an odorant reinforced with an electric shock (black squares) and a non-reinforced odorant (white squares) during 12 trials (6 reinforced and 6 non-reinforced). Bees learned to discriminate between odorants as a result of conditioning. *: p<0.0001.

To determine whether SER can be conditioned using olfactory stimuli, we trained bees with 6 explicitly paired presentations of an odorant and the electric shock. The interstimulus interval (interval between odorant and shock onset) was 3 sec and the intertrial interval was 10 min. We compared the performance of these bees to that of bees trained with explicitly unpaired presentations of odorant and shock. Bees of the unpaired group experienced 6 odorant and 6 shock presentations that were temporally dissociated. In all cases we recorded SER to the odorant. Each group was subdivided into two subgroups trained either with 1-hexanol or with eugenol. One hour after the last trial bees were presented with the conditioned odorant alone in a retention test. The two paired subgroups (1-hexanol, eugenol) did not differ significantly and were therefore pooled, as were the two unpaired subgroups. Over successive trials, bees from the paired group (n = 38) significantly increased their response to the odorant that preceded the electric shock (Fig. 2a; ANOVA for repeated measurements: F_5,190_ = 8.46, p<0.0001). Bees in the unpaired group (n = 39) showed no significant change in responsiveness to the unpaired odorant (F_5,185_ = 2.19, NS) during trials, thus demonstrating that the increase in SER observed in the paired group was due to associative learning and not to the simple experience with the odorant and the shock, independent of their temporal sequence. The performance of both groups differed significantly over all trials (F_1,75_ = 18.35, p<0.0001) and the evolution of responses during trials was also different between groups, as shown by the significant interaction (F_5,375_ = 9.79, p<0.0001). One hour after conditioning, bees of the paired group still remembered the conditioned odorant while bees of the unpaired did not respond to the odorant (Fig. 2a, black and white bars, respectively; Fisher's exact test; p<0.0001). An aversive memory was therefore established in the paired but not in the unpaired group.

To confirm the associative nature of this learning, we trained bees to extend their sting to an odorant paired with an electric shock and not to respond to a non-reinforced odorant (differential conditioning). This procedure is a typical within-subject control in studies of associative learning. Bees were conditioned during 6 reinforced and 6 non-reinforced trials, presented in a pseudo-random sequence with 10 min inter-trial intervals. For one group of bees, eugenol was the reinforced odorant and 1-hexanol the non-reinforced odorant; for a second group the contingencies were inversed. Because there were no significant differences between both groups, data were pooled (n = 48). The resulting learning curves (Fig. 2b) show that bees learned to discriminate between odorants as a result of conditioning (F_1,94_ = 27.05, p<0.0001). Thus, olfactory conditioning of SER is truly associative and does not depend on the simple exposure to the training stimuli, independent of their outcome.

**Figure 3:**
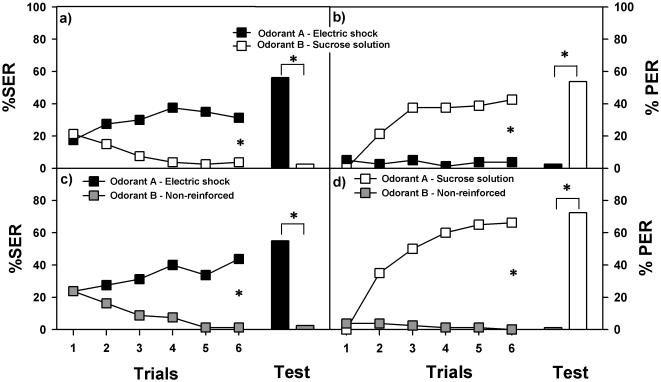
Simultaneous aversive and appetitive learning in honeybees. The same group of bees (SER-PER group; n = 80) was trained in a double discrimination task with an odorant (‘A’) paired with an electric shock that elicited the sting extension reflex (SER) and with another odorant (‘B’) paired with sucrose solution delivered to the antennae and proboscis that elicited the proboscis extension reflex (PER). a) Bees responded significantly with a SER to the odorant associated with the electric shock (black dots and bar), but not to that associated with sucrose (white dots and bar). b) The same bees responded significantly with a PER to the odorant associated with sucrose (white dots and bar), but not to that associated with electric shock (black dots and bar). One hour after the last conditioning trial, bees still responded correctly to the odorants (bars), even if their respective USs were absent in the tests. As a result of training, bees exhibited the appropriate response to the appropriate odorant. Appetitive and aversive learning can thus be mastered simultaneously. c,d: Control groups trained to discriminate odorants A and B, one of which was non-reinforced and the other reinforced either with electric shock (c: SER group; n = 80) or with sucrose solution (d: PER group; n = 80). c) Bees responded significantly with a SER to the odorant associated with the electric shock (black squares and bar), but not to the non-reinforced odorant (grey squares and bar). The performance of this group did not differ from that of the SER-PER group [compare with a)]. d) Bees responded significantly with a PER to the odorant associated with the sucrose solution (white squares and bar), but not to the non-reinforced odorant (grey squares and bar). The performance of this group was significantly better than that of the SER-PER group [compare with b)]. *: p<0.0001.

We then asked whether or not appetitive and aversive olfactory learning can be mastered simultaneously by honeybees. We trained bees to discriminate 1-hexanol and 1-nonanol, one of which was paired with electric shock during 6 trials and the other with sucrose solution 50% (weight/weight) also during 6 trials (SER-PER group). Trials were spaced by 10 min and odorants were presented in a pseudo-random sequence. Data from both conditioned groups (1-hexanol/shock vs. 1-nonanol/sucrose and 1-hexanol/sucrose vs. 1-nonanol/shock) could be pooled (n = 80). The resulting performance is presented in Fig. 3a,b. Bees responded significantly with a SER to the odorant associated with the electric shock but not to that associated with sucrose (Fig. 3a: F_1,158_ = 27.33, p<0.0001) whereas they responded significantly with a PER to the odorant associated with sucrose but not to that associated with electric shock (Fig. 3b: F_1,158_ = 40.89, p<0.0001). As a result of training, they exhibited the appropriate response to the appropriate odorant. One hour after the last conditioning trial, bees still responded correctly to the odorants even in the absence of punishment or reward (McNemar test; SER: χ^2^ = 42.00; p<0.0001; PER: χ^2^ = 37.21; p<0.0001).

To show that bees did indeed learn to master both appetitive and aversive associations simultaneously, we focused on individual performances and quantified the number of bees responding correctly to *both* the aversive and the appetitive odorants (‘doubles’), to the appetitive odorant alone (‘*PER only*’) and to the aversive odorant alone (‘SER only’). As bees were naïve for both odorants in the first trial, we included only responses from trial 2 to 6 in our analysis. Only the number of bees responding correctly to the aversive *and* to the appetitive odorant (‘doubles’) increased significantly from trial 2 to 6 (from 2.5 to 13.75%; F_4,316_ = 4.42; p<0.05). Neither the number of bees responding correctly *only* to the odorant paired with sucrose (‘PER only’: from 18.75 to 28.75%; F_4,316_ = 1.73; NS) nor the number of bees responding correctly *only* to the odorant paired with electric shock (‘SER only’: from 25 to 17.5%; F_4,316_ = 1.34; NS) varied significantly. For these bees, ‘PER only’ and ‘SER only’, the significant increase occurred between the first and second trial. Thus, at the end of conditioning, 13.75% of bees (11 out of 80 bees) mastered both associations. In the retention tests, the proportion of ‘doubles’ responding correctly to both the aversive and the appetitive odorant was even higher (30%; 24 out of 80 bees), thus suggesting that additional trials and/or time improves the performance of bees in this double task. The proportion of ‘PER only’ bees in the retention test was 23.75% (19 out of 80 bees) and that of ‘SER only’ bees 27.5% (22 out of 80 bees).

In parallel to the double-task discrimination shown in Fig. 3a,b we trained two groups of bees to discriminate the same odorants, 1-hexanol and 1-nonanol, either in the SER (Fig. 3c) or in the PER protocol (Fig. 3d). These groups had to learn a single discrimination task, aversive or appetitive, and were conceived to estimate whether mastering of both appetitive and aversive associations resulted in an impaired performance in either discrimination. In the SER group, one odorant was associated with electric shock during 6 trials while the other was presented without punishment during 6 trials. In the PER group, bees were fixed in the same holders used for SER conditioning and they experienced one odorant associated with sucrose solution during 6 trials and another non-rewarded odorant during 6 trials. For each group, odorants were balanced and data from bees trained with 1-hexanol and 1-nonanol could be pooled (PER group: n = 80; SER group: n = 80). Trials were spaced by 10 min and odorants were presented in a pseudo-random sequence as in the group that learned both associations simultaneously. As shown previously (see Fig. 2b), bees learned to discriminate one odorant reinforced with electric shock from an unpunished odorant so that they extended their sting to the reinforced but not to the non-reinforced odorant (Fig. 3c: F_1,158_ = 31.41, p<0.0001). Similarly, bees learned to discriminate one odorant rewarded with sucrose solution from a non-rewarded odorant so that they extended their proboscis to the rewarded but not to the non-rewarded odorant (Fig. 3d: F_1,158_ = 128.08, p<0.0001). One hour after the last conditioning trial, bees in each group responded correctly to the odorants even in the absence of punishment (SER group: χ^2^ = 36.54, p<0.0001) or of reward (PER group: χ^2^ = 55.02, p<0.0001). We then compared the performance of these groups (Fig. 3c,d) to that of the SER-PER group trained in parallel with both aversive and appetitive USs (Fig 3a,b). Learning both aversive and appetitive associations did not affect aversive SER conditioning as the level of SER responses was identical for the SER-PER group (Fig. 3a) and the SER group (Fig. 3c), both for acquisition (F_1,306_ = 0.31, NS) and retention (Mann-Whitney test performed on the difference between responses to test odorants: Z = 0.12, NS). However, learning both associations resulted in a lower performance for appetitive PER conditioning. Bees in the SER-PER group reached lower levels of PER acquisition and retention compared to the PER group which did not experience electric shocks (acquisition: F_1,316_ = 7.10, p<0.01; retention: Z = 2.48, p<0.02). Although aversive learning seems to interfere with appetitive learning, lower PER performances in the SER-PER group may simply result from having experienced six electric shocks that would lower the general responsiveness of the bees without implying necessarily interferences between different forms of associative learning.

To test this hypothesis we performed an additional control experiment in which we explicitly tested the effect of the electric shock on appetitive PER conditioning. We trained bees to respond to an odorant, either 1-hexanol or 1-nonanol, paired with sucrose solution, and interspersed the shock alone during training. Thus, bees experienced six paired presentations of odorant and sugar reward and six electric shocks following the same pseudorandom sequence as in the previous experiments (‘shock group’). In a parallel group, the same procedure was followed but the shock was substituted by a placement trial in which bees were simply placed in the conditioning setup without receiving any punishment (‘placement group’). One hour after training, bees of both groups were presented in a retention test with the trained odorant and with a novel odorant (1-hexanol for bees trained with 1-nonanol and vice versa) in order to assess the specificity of olfactory memory. As in the previous experiments, trials were spaced by 10 min. The odorants, sucrose concentration and shock intensity were the same as before.

**Figure 4:**
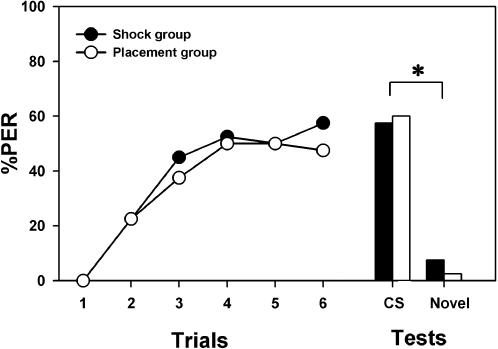
The effect of electric shock on appetitive olfactory conditioning of the proboscis extension reflex (PER). Responses (PER) of bees trained to associate an odorant with sucrose solution along six conditioning trials and experiencing six electric shocks (‘shock group’; black dots and bars; n = 40) or six placements in the conditioning setup (‘placement group’; white dots and bars; n = 40) interspersed pseudorandomly. Bees in both groups learned to respond to the rewarded odorant irrespective of the presence or absence of shock. One hour later, both groups behaved similarly in the retention test (bars) and responded significantly more to the conditioned odorant than to the novel odorant. Thus, repetitive stimulation with the electric shock did neither affect appetitive olfactory learning nor olfactory memory one hour after conditioning. *: p<0.0001.

For each group, ‘shock’ and ‘placement’, the two subgroups conditioned to either 1-hexanol or 1-nonanol yielded similar results and were therefore pooled (group shock: n = 40; group placement: n = 40). Both groups of bees learned to respond to the rewarded odorant during training, irrespective of the presence or absence of electric shocks during conditioning (Fig. 4; F_5,390_ = 33.18, p<0.0001). The acquisition was similar in both groups (F_1,78_ = 0.20, NS) and no significant interaction was found (F_5,390_ = 0.36, NS). During the retention test, bees of both groups behaved similarly (Z = 0.47, NS) as they responded significantly more to the learned odorant than to the novel odorant (χ^2^ = 39.20; p<0.0001), thus showing that the shocks experienced during training did not affect the appetitive memory one hour after conditioning. These results show, therefore, that the electric shock did not interfere with appetitive olfactory learning. We conclude from these experiments, that while the formation of an appetitive association does not interfere with aversive conditioning, the formation of an aversive odorant-shock association (*and not the shock alone*) induces a performance decrease during appetitive conditioning. However, the fact that some bees manage to learn both associations simultaneously supports the notion that appetitive and aversive olfactory learning are mediated by relatively independent neural systems (also termed ‘modules’) dedicated to the processing of appetitive and aversive associations.

Looking at the neural bases of appetitive and aversive olfactory learning and studying whether the appetitive and aversive unconditioned stimuli used are mediated by different neurotransmitters may help elucidate the relative independence of appetitive and aversive modules. We took advantage of the fact that in our aversive learning protocol bees are immobilized, thus allowing simultaneous access to behavioral performances and to the nervous system. We tested whether octopamine and dopamine, two catecholamines that have been related respectively to appetitive and aversive olfactory learning in fruit flies [18], [19] and crickets [20] are required for olfactory aversive learning in honeybees. In honeybees, octopamine mediates sucrose reward in olfactory appetitive learning [21], [22] but nothing was known about aversive learning because the possibility of studying aversive learning at the neural level was so far unavailable. Ringer solution (control), octopaminergic (mianserine 0.33 µM or 3.3 mM, epinastine 4 mM) or dopaminergic receptor antagonists (fluphenazine 0.19 µM or 1.9 mM, flupentixol 0.2 µM or 2 mM) were injected (10×20 nl in all cases) into the brain through the median ocellar tract 30 min before conditioning. We then trained bees to discriminate 1-hexanol and eugenol using the differential conditioning procedure described above (see Figs. 2b and 3c). Ringer controls did neither differ for acquisition (Kruskal-Wallis test: H_2,104_ = 8.48; NS) nor for retention (H_2, 102_ = 2.30; NS). We therefore analyzed the performance of drug-injected groups and compared it to that of Ringer-injected groups.

**Figure 5:**
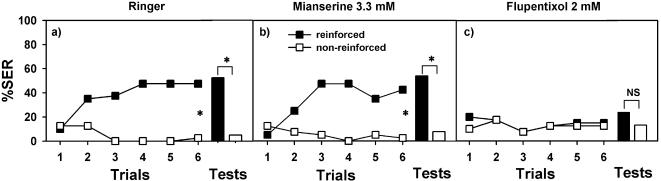
The effect of octopaminergic and dopaminergic receptor antagonists on olfactory conditioning of the sting extension reflex (SER). Responses (SER) of bees trained to discriminate an odorant reinforced with an electric shock (black squares) and a non-reinforced odorant (white squares) during 12 acquisition trials (6 reinforced and 6 non-reinforced). A retention test was conducted 1 h after the last acquisition trial (black bar: odorant previously reinforced; white bar: odorant previously non-reinforced). a) Responses (SER) of control bees injected with Ringer into the brain (n = 40); b) Responses (SER) of bees injected with the octopaminergic antagonist mianserine 3.3 mM into the brain (n = 40); c) Responses (SER) of bees injected with the dopaminergic antagonist flupentixol 2 mM into the brain (n = 40). Ringer-and mianserine-injected bees learned to discriminate the reinforced from the non-reinforced odorant and remembered the difference one hour later. Flupentixol-injected bees did not learn to discriminate the reinforced from the non-reinforced odorant, nor did they respond appropriately in the retention tests. Similar results were obtained with other concentrations of octopaminergic and dopaminergic antagonists (see Fig. 6). These results show that dopamine, but not octopamine receptors are required for aversive olfactory learning in honeybees.

**Figure 6:**
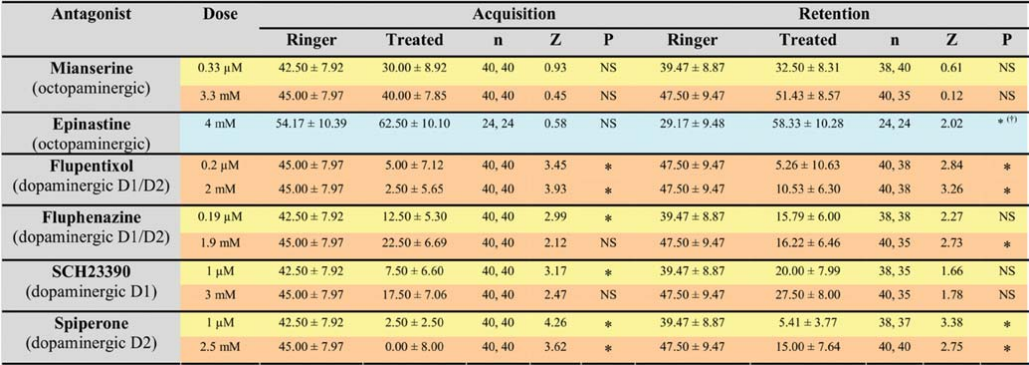
The effect of different octopaminergic and dopaminergic antagonists on acquisition (differential conditioning with two odorants, one reinforced and the other non-reinforced) and retention (memory test 1h after conditioning) of olfactory aversive learning in honeybees. Values correspond to the mean±S.E of a discrimination index (responses to reinforced odorant–responses to non-reinforced odorant) in the last acquisition trial and in the retention test. They are expressed in%. Sample sizes are indicated in parentheses. Colors correspond to the groups that were performed in parallel. Groups sharing the same color share the same Ringer control. Pairwise comparisons between drug-and Ringer-injected (control) groups were performed using a Mann-Whitney test. Z-adjusted values and significance level (p) are given for each comparison. NS: non-significant; *: p<0.05 in blue row; *: p<0.0125 in yellow rows (α/4); *: p<0.008 in pink rows (α/6). (^†^): Significance in this case is due to an unusually low performance in the control (Ringer-injected) group, and not to an incremental effect of epinastine.

Ringer-injected bees learned to discriminate the reinforced from the non-reinforced odorant (see performance of one of the three Ringer controls in Fig. 5a: F_1,39_ = 67.49, p<0.0001). One hour later, they still remembered the aversive association and extended their sting in response to the previously punished odorant (McNemar Test: χ^2^ = 9.09, p<0.01). Octopaminergic antagonists (mianserine or epinastine) did not affect performance at any of the concentrations used in these experiments (Fig. 6). Similarly to Ringer-injected bees, mianserine-and epinastine-injected bees learned to discriminate the two odorants and responded with SER only to the odorant paired with the electric shock (mianserine 0.33 µM: F_1,39_ = 27.42, p<0.0001; mianserine 3.3 mM: F_1,39_ = 60.14, p<0.0001; epinastine 4 mM: F_1,23_ = 50.76, p<0.0001). Retention tests also showed significant discrimination (mianserine 0.33 µM: χ^2^ = 7.58, p<0.01; mianserine 3.3 mM: χ^2^ = 12.04, p<0.001; epinastine 4 mM: χ^2^ = 12.07, p<0.001). An apparent increase of retention was found in bees injected with epinastine with respect to Ringer-injected bees (see light blue row in Fig. 6) but this effect was due to the poor performance of Ringer-injected bees rather than to epinastine itself. Figure 5b shows as example the performance of bees injected with mianserine 3.3 mM, which learned to discriminate the punished from the non-punished odorant and remembered the difference one hour later. Thus, octopaminergic antagonists did not impair aversive olfactory learning in honeybees.

Dopaminergic antagonists (fluphenazine and flupentixol) had a dramatic effect on aversive olfactory learning. For both concentrations of flupentixol tested, and contrarily to controls, bees did not learn to discriminate between odorants (flupentixol 0.2 µM: F_1,39_ = 0.88, NS; flupentixol 2 mM: F_1,39_ = 0.78, NS). Consequently, they did not show discrimination in the tests performed one hour later (flupentixol 0.2 µM: χ^2^ = 0.64, NS; flupentixol 2 mM: χ^2^ = 0.06; NS). As an example, Figure 5c presents the performance of bees injected with flupentixol 2 mM, which learned neither to discriminate the reinforced from the non-reinforced odorant nor to respond appropriately in the retention test. For fluphenazine, significant differences with the Ringer control were found in two out of four comparisons (see Fig. 6) and the alpha value of the two other comparisons was close to significance. These results show therefore that dopamine-, but not octopamine signaling, is necessary for aversive olfactory learning in honeybees.

**Figure 7:**
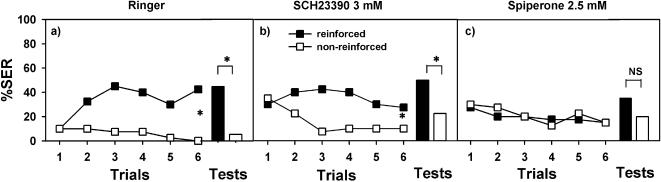
The effect of vertebrate D1-like and D2-like dopamine receptor antagonists on olfactory conditioning of the sting extension reflex (SER). Responses (SER) of bees trained to discriminate an odorant reinforced with an electric shock (black squares) and a non-reinforced odorant (white squares) during 12 acquisition trials (6 reinforced and 6 non-reinforced). A retention test was conducted 1 h after the last acquisition trial (black bar: odorant previously reinforced; white bar: odorant previously non-reinforced). a) Responses (SER) of control bees injected with Ringer into the brain (n = 40); b) Responses (SER) of bees injected with the D1-like dopamine receptor antagonist SCH23390 3 mM into the brain (n = 40). c) Responses (SER) of bees injected with the D2-like dopamine receptor antagonist spiperone 2.5 mM into the brain (n = 40). Ringer-and SCH23390-injected bees learned to discriminate the reinforced from the non-reinforced odorant and remembered the difference one hour later. Spiperone-injected bees did not learn to discriminate the reinforced from the non-reinforced odorant, nor did they respond appropriately in the retention tests. These results suggest that *Am*DOP receptors could contribute differently to aversive learning in bees.

In vertebrates, dopaminergic receptors are generally classified in two main families, the D1-like and D2-like receptors [23], [24]. Activation of the D1-like family is coupled to increases in cAMP concentration and is typically excitatory, while D2-like activation reduces cAMP and is typically inhibitory. In the honeybee, three different dopamine receptors have recently been identified: *Am*DOP1 [25], *Am*DOP2 [26] and *Am*DOP3 [27]. *Am*DOP1 and *Am*DOP3 have been related to the vertebrate D1-like and D2-like family of dopamine receptors, respectively [25], [27]. *Am*DOP2 appears to be more closely related to invertebrate octopamine receptors and constitutes therefore a distinct ‘invertebrate type’ dopamine receptor [26]. From a functional point of view, however, it can be referred to as a ‘D1-like receptor’ because it upregulates cAMP. Contrarily to vertebrates, no specific pharmacological blockers for D1-like and D2-like receptors are yet available in insects. We nevertheless used vertebrate D1-like and D2-like receptor blockers SCH23390 and spiperone, respectively, to confirm that dopamine is necessary for aversive olfactory learning in honeybees and to determine whether these drugs affect differently acquisition and retention in our protocol. We followed the procedure described above and injected either Ringer (control), SCH23390 (1 µM and 3 mM) or spiperone (1 µM and 2.5 mM) into the brain. Results are given in Fig. 6. Ringer-injected bees (n = 40) learned to discriminate the reinforced from the non-reinforced odorant (F_1,39_ = 54.65, p<0.0001). One hour later, they still remembered the difference (χ^2^ = 10.56, p<0.005). Figure 7a shows as example the performance of one of the two Ringer control conditioned in parallel with the SCH23390 and the spiperone groups.

SCH23390-injected bees failed to show evidence of acquisition at the low dose (1 µM: F_1,39_ = 2.77, NS) but not at the high dose (3 mM: F_1,39_ = 23.15, p<0.005) (n = 40 in both cases). SCH23390 had no effect on retention as bees responded significantly more to the odorant previously paired with the electric shock and did not differ from control bees (1 µM: χ^2^ = 4.00, p<0.05; 3 mM: χ^2^ = 10.08, p<0.002). Figure 7b shows as example the performance of the group injected with 3 mM of SCH23390. Taken together, these results indicate that SCH23390 had a low detrimental effect on aversive learning in bees.

By contrast, spiperone-injected bees were significantly impaired in both learning and retention at both doses used (n = 40 in both cases) as they did not learn the difference between reinforced and non-reinforced odorants (1 µM: F_1,39_ = 2.75, NS; 2.5 mM: F_1,39_ = 0.19, NS) nor did they show any retention one hour later (1 µM: χ^2^ = 0.50, NS; 2.5 mM: χ^2^ = 2.90, NS). Figure 7c shows as example the performance of the group injected with 2.5 mM of spiperone. These results show that olfactory aversive learning is differently affected by blockade by different dopaminergic ligands. Although specific D1-like and D2-like dopamine receptor blockers are still required for insects, our results suggest that *Am*DOP receptors could contribute differently to aversive learning in bees.

## Discussion

The present work shows that harnessed bees learn to associate odorants and electric shock in the laboratory and that it is possible to access the bee brain while the animal learns and memorizes aversive cues. Our protocol allows associative, aversive learning to be studied at the individual level, in a framework that is distinct from any appetitive behavior. Previous studies focused on avoidance learning in foraging bees and preserved therefore an appetitive framework [28]–[32]. In such studies, free-flying bees foraging for food learned to avoid flower patches infested with crab spiders [28], [29] or artificial flowers penalized either with quinine [30] or a puff of compressed air [31]. They also learned to avoid landing on five out of six petals of a mechanical flower that flicked forward and hit them upon landing [32]. Electric shock has also been used, although seldom, to generate avoidance of visited food sources in free-flying honeybees [33], [34]. All these studies have in common the impossibility of accessing the nervous system in parallel to behavioral recording because they used free-flying bees. Furthermore, they all maintain an appetitive framework as they aim to inhibit the appetitive response of food search. The appetitive framework is also present in a variant of olfactory PER conditioning in which after pairing an odorant and sucrose, an electric shock is delivered to the proboscis so that bees learn to retract it in response to the odorant [35]. Our assay, in contrast, precludes confounding appetitive responses and converges on experimental conditioning procedures traditionally used for *Drosophila* in which odorants are directly paired with electric shock [12] without involving an appetitive context. In the fruit fly, this assay led to significant progress in the study of learning and memory and its importance has been underlined [36]. Our procedure has, however, a significant advantage with respect to olfactory conditioning in *Drosophila* as it does not contain orienting or locomotion components, which could be interpreted as an operant component in an otherwise classical conditioning paradigm. We expect, therefore, that our assay will facilitate new research and comparative studies on the neurobiology of aversive learning and memory, which have up to now been impossible in honeybees.

We showed that bees trained in a double discrimination task (one odorant rewarded with sucrose solutions vs. a different odorant punished with electric shock) learn to master both appetitive and aversive olfactory learning simultaneously, so that they exhibit the appropriate response, PER or SER, to the appropriate odorant. Bees trained in such a way exhibit, however, lower scores for appetitive learning than bees trained to discriminate two odorants in the appetitive modality (one odorant rewarded with sucrose solutions vs. a different odorant non-rewarded). This difference was not due to a detrimental effect of the electric shock as shown by the fact that appetitive olfactory learning was unaffected by interspersing shocks among odorant-sugar trials. The lower performance in appetitive conditioning (PER) found in bees trained with the double discrimination task (Fig 3b) could be due to the potential difficulty of actively producing two different motor responses, PER and SER. Indeed, in the PER-alone group (Fig 3d), bees only had to respond to odorants with one response, PER. However, if this were the case, it should have produced a similar detrimental effect onto SER conditioning in the double discrimination group relative to the SER-alone group, which only had to respond to odorants with one response, SER. This was not the case. Thus, although the fact that some bees manage to learn both tasks simultaneously underlines the relative independence of aversive and appetitive olfactory learning in honeybees, there seems to be a negative influence of the formation of an odorant-shock association on simultaneous appetitive conditioning. A possible explanation for this effect could relate to a natural predisposition of the bee brain to give priority to the aversive/defense system relative to the–less critical-appetitive system in cases of threatening situations.

To determine whether the relative independence of appetitive and aversive learning is supported by independent modules corresponding to separate neural systems dedicated to the processing of the different unconditioned stimuli, we took advantage of the possibility of combining aversive conditioning and neuropharmacological tools. Octopamine has been strongly implicated in appetitive olfactory learning in bees and octopamine injections in the brain can substitute for sucrose reward and induce olfactory learning [21]. Blocking octopamine receptors by means of RNAi techniques disrupts olfactory conditioning of PER [22]. In addition, we found that dopamine, but not octopamine, underlies aversive olfactory learning, thus suggesting that dopamine is linked to aversive learning across insect species [18]–[20]. Our results suggest that dopamine plays an instructive function in aversive learning, possibly conveying information about punitive stimuli. Dopaminergic neurons capable of mediating and predicting aversive stimuli have been found in the *Drosophila* brain [37]. These neurons may be a general feature of the insect brain and dopamine may underlie other forms of aversive learning involving stimuli of different sensory modalities (e.g. *visual* stimuli associated with aversive *gustatory* USs [38]). We suggest that the activity of modulatory, aminergic neurons serves, in the insect species tested so far, as a value system in associative learning phenomena, i.e. as a system allowing ordering, prioritizing and assigning appropriate labels to learnt stimuli [39].

We also showed that differences in learning and retention can be found on the basis of their susceptibility to blocking by dopaminergic ligands. The ligands used are well-known vertebrate D1-like and D2-like receptor antagonists. Although the specificity of these antagonists is not granted for insect receptors, the fact that the D1-like antagonist SCH23390 was less effective than the D2-like antagonist spiperone, which suppressed aversive learning and memory, raises the question of the differential involvement of *Am*DOP receptors in aversive learning and memory in bees. Studying these questions will require, however, the development of specific pharmacological antagonists or the use of RNAi techniques already applied successfully in honeybees [22].

For honeybees, stinging can eventually lead to death because the sting may remain attached to the stung surface [13]. Nevertheless, we show here that the stinging response can be conditioned, thus underlining the remarkable plasticity of the insect nervous system. In a natural context, bees do not always die when they sting an enemy, especially if it offers soft body mass and non-elastic surfaces which allow the sting to retract [13]. This is the case for predators like wasps and other insects, which commonly attack hives and elicit defensive behavior without resulting in sting loss. Olfactory discrimination is critical for enemy identification in defending bees [13]. It may thus be adaptive to learn to recognize predators and exhibit appropriate defensive behavior in an aversive context.

Finally, we discuss the pertinence of the terminology used to characterize our conditioning protocol. We have defined the olfactory conditioning of the sting extension reflex as a case of aversive learning. Contrarily to other cases of aversive learning, however, the consequence of SER conditioning is neither an avoidance response towards the stimulus (here a given odorant) predicting the noxious unconditioned stimulus (here the shock) nor a response inhibition. In traditional protocols of aversive learning, individuals learn to actively avoid a noxious stimulus (e.g. fruit flies trained to associate an odorant with an electric shock in a T-maze actively avoid the arm of the maze presenting the odorant, which was negatively reinforced [12]) or to inhibit a response when confronted with a potentially harmful situation (e.g. mice trained to associate a given context with an electric shock exhibit a freezing response when replaced in the same context [40], [41]). In SER conditioning, bees learn to redirect an active response, stinging, towards an originally neutral stimulus that predicts shock delivery. This difference does not invalidate the term ‘aversive’ used to characterize our protocol but underlines the importance of relating responses to their biological background. In a natural context, bees facing a potential danger are not supposed to escape but to attack. In that sense the natural response to an aversive stimulus is precisely what is recorded in our protocol. We thus maintain that, independently of the fact that bees actively produce a response instead of inhibiting it, SER conditioning is a case of true aversive learning as it relies on the relevant, natural response to an aversive stimulus.

## Materials and Methods

### Olfactory conditioning of SER

Honeybees were captured at the entrance of an outdoor hive. These individuals, which are usually older than 5–7 days, have a fully developed sting reflex [15]. Bees were chilled on ice for 5 min until they stopped moving. Each bee was individually fixed on a metal holder (Fig. 1) consisting of two brass plates (E1, E2) fixed to a Plexiglas plate (pp). The bee's petiole was tightly fitted into the notch N2 and the neck into the notch N1. The elastic girdle G clamped the thorax. Plates E1 and E2 were connected to the output of the stimulator (60 Hz–AC current). Notches N1 and N2 were smeared with an EEG gel (Spectra 360 Electrode Gel, Parker Laboratories) to ensure good contact between the plates and the bee. The resistance measured between E1 and E2 in the presence of the bee was 200–300 KΩ. The aversive US was an electric shock of 7.5 V applied for 2 sec. Five µl of pure odorants (1-hexanol, eugenol and 1-nonanol, Sigma Aldrich, Deisenhofen, Germany) were applied onto 1 cm^2^ filter paper pieces which were transferred to 20 ml syringes allowing odorant delivery to the antennae. Each odorant was delivered for 5 sec. An air extractor placed behind the bee prevented odorant accumulation, as well as possible contamination by pheromone release.

Each conditioning trial lasted 1 min. The bee was placed in the stimulation site in front of the air extractor and left for 20 sec before being exposed to the odorant paired with the electric shock. The electric shock started 3 sec after odorant onset and finished with the odorant. The bee was then left in the setup for 35 sec and then removed. The intertrial interval (ITI) was always 10 min. Retention tests were performed 1 h after the last conditioning trial and consisted of presenting odorant stimuli without punishment. In order to ensure an ITI of 10 min, groups of 10 bees were trained one after the other. In this way, 10 min were required for the bees to complete each trial and to move to the next trial. Several conditioning apparatuses were available to run several groups in parallel if necessary.

In the paired group (Fig. 2a), conditioning trials were alternated with 6 blank trials. During a blank trial the bee was placed in the experimental position for 1 min and no specific stimulus was delivered. Blank trials were used in the paired group to equate the number of contextual experiences between paired and unpaired groups. In the unpaired group (Fig. 2a), trials lasted also 1 min, and only the odorant or the electric shock was presented. Thus, bees of both groups, paired and unpaired, were subjected to 6 odorant stimulations, 6 electric shocks stimulations and 12 placements, so that only the explicit pairing of odorant and shock differed between groups. In differential conditioning (Fig. 2b) odorants were presented in a pseudo-random sequence of 6 reinforced and 6 non-reinforced trials (e.g. ABBABAABABBA) starting with odorant A or B in a balanced presentation.

Experiments combining appetitive PER and aversive SER conditioning (Fig. 3a,b) were performed with bees fixed in the holders used for electric shock delivery. Odorants were presented in a pseudo-random sequence of 6 sucrose-reinforced and 6 electric-shock-reinforced trials (see above). Trials reinforced with 50% sucrose solution followed the same schedule as trials reinforced with electric shock (5 sec odorant and 2 sec sucrose to the antenna and then to the proboscis, starting 3 sec after the odorant onset). Control groups trained in the aversive (Fig. 3c) or the appetitive (Fig. 3d) modality were prepared and treated as the group receiving the combined aversive and appetitive USs but received only one kind of US, electric shock or sucrose solution.

In all experiments, US responses were measured before and after conditioning or retention test. Only bees which consistently reacted to the electric shock were taken into consideration. This is particularly important when testing the effect of drug injection (see below) as it precludes the possibility that the analysis reflects the action of a given antagonist on a pathway controlling only the sting extension reflex, irrespective of the possible association with the paired odorant.

### Neuropharmacological experiments

Drugs were dissolved in honeybee Ringer [NaCl 130 mM, KCl 6 mM, MgCl_2_ 4 mM, CaCl_2_ 5 mM, HEPES 10 mM, glucose 25 mM, sucrose 160 mM]. Drugs or Ringer alone (control) were injected into the brain through the median ocellar tract. In addition to normal preparation, the head of each harnessed bee was fastened to the holder with a small drop of wax to avoid movements. A Harvard GC 100-10 microelectrode filled with the drug to be injected was connected to an IM 300 Narishige microinjector and used to deliver 10×20 nl into the brain. Volumes injected were calibrated before and after injection by means of a Malassez cell. Injections were performed 30 min before conditioning, as earlier experiments (reviewed in ref. 42) showed that pharmacological injections of catecholamines and their inhibitors are effective approximately 30 min after drug application. Octopaminergic (mianserine, epinastine) and dopaminergic receptor antagonists (fluphenazine, flupentixol, SCH23390, spiperone) (Sigma Aldrich, Deisenhofen, Germany) were used at µm and mM doses (Fig. 6). Low-dose experiments (except for flupenthixol 0.2 µm) and their respective Ringer control were performed in parallel (yellow rows in Fig. 6). High-dose experiments (and flupentixol 0.2 µm) were also performed in parallel with their respective Ringer control (pink rows in Fig. 6). The group injected with epinastine received one dose and had a separate Ringer control (blue row in Fig. 6).

### Statistics

During the experiments, we recorded the response to the presented odorant, i.e. whether bees extended their sting after the onset of the odorant and before the presentation of the electric shock in the case of reinforced trials so that conditioned responses were recorded. Multiple responses during odorant presentation were counted as a single SER. The percentage of SER recorded during acquisition was used to plot acquisition curves. Similar procedure was followed for PER in the case of appetitive learning. To analyze the variation of performance during trials, we used analyses of variance (ANOVAs) for repeated measurements both for between-group and for within-group comparisons. Monte Carlo studies have shown that it is permissible to use ANOVA on dichotomous data only under controlled conditions [43], which are met by our experiments (equal cell frequencies and at least 40 degrees of freedom of the error term). Performances within a retention test were analyzed by means of a McNemar test. To compare drug-injected bees and their respective Ringer controls, we calculated for each group a *discrimination index* (values reported in Fig. 6), which was the difference between the responses to the reinforced odorant minus the responses to the non-reinforced odorant. This differential index (in%) was calculated for the last acquisition trial (6^th^ trial), as it constitutes an appropriate measure of the discrimination reached at the end of conditioning, and for the retention test. Comparisons between groups were done using a Mann-Whitney test. Bonferroni corrections were used to adjust the alpha levels of comparisons involving groups performed in parallel.
